# Microbiological evidence for the trisubstituted benzimidazoles targeting MmpL3 in *Mycobacterium tuberculosis*

**DOI:** 10.1128/aac.00368-25

**Published:** 2025-08-19

**Authors:** Mengyun Zhang, Renee Allen, Lauren Ames, Curtis A. Engelhart, Diana Quach, Xiaoying Lv, Genhui Xiao, Heng Wang, Jinglan Wang, Liangliang Zhou, Miaomiao Pan, Joseph Sugie, Joe Pogliano, Dirk Schnappinger, Tanya Parish, Shawn Chen

**Affiliations:** 1 Global Health Drug Discovery Institute 707154, Beijing, China; 2 Center for Global Infectious Disease Research, Seattle Children's Research Institute 549448 , Seattle, Washington, USA; 3 Department of Microbiology and Immunology, Weill Cornell Medicine 12295, New York, New York, USA; 4 Linnaeus Bioscience Inc. 721469 , San Diego, California, USA; 5 Department of Pediatrics, University of Washington School of Medicine 12353 , Seattle, Washington, USA; Bill & Melinda Gates Medical Research Institute, Cambridge, Massachusetts, USA

**Keywords:** *Mycobacterium tuberculosis*, MmpL3, mechanisms of action

## Abstract

New anti-tuberculosis (TB) drugs with novel modes of action are in great demand due to the complex treatment regimens as well as the rising number of multidrug-resistant TB cases. We recently re-evaluated a few 2,5,6-trisubstituted benzimidazole derivatives (SBZ) previously demonstrated to have potent antitubercular activity. These compounds displayed favorable MICs and significantly reduced bacterial counts in an acute mouse infection model. Although this antitubercular lead series was initially reported to inhibit mycobacterial cell division, our findings suggest that its primary activity likely involves other cellular targets. By using bacterial cytological profiling, we observed that SBZ-treated *Mycobacterium tuberculosis* cells exhibit cell wall-damaging phenotypes resembling those caused by known cell wall biosynthesis inhibitors, such as AU1235 and SQ109, that mostly target the membrane protein large 3 (MmpL3). Whole-cell assays further supported the findings by showing activation of the *iniBAC* operon and accumulation of intracellular ATP. The antitubercular activity of SBZs was tested against engineered mycobacterial strains that have the transcriptionally regulated *mmpL3* gene expression, confirming that SBZs engage the MmpL3 target in the cell. Strains with mutations in *mmpL3* exhibited either low- or high-level resistance to the SBZs. A molecule docking model is proposed, based on a high-resolution crystal structure of MmpL3, which could be useful in reconciling the inhibition mechanism and suggesting a further development of MmpL3 inhibitor starting with the SBZ scaffold.

## INTRODUCTION

Tuberculosis (TB), caused by a single etiological agent, *Mycobacterium tuberculosis* (Mtb), remains a critical global health challenge, with over 8.2 million new cases and 1.25 million deaths reported in 2023 (Global Tuberculosis Report 2024, WHO). The increasing prevalence of multidrug-resistant TB underscores the urgent need for novel therapeutics that target previously untapped pathways. High-throughput whole-cell screening has become instrumental in identifying phenotypic hits active against Mtb. The approach often leads to the discovery of compounds that disrupt essential cellular processes, such as cell wall biosynthesis and energy metabolism (1, 2). Among these, MmpL3, a transporter required for trehalose monomycolate (TMM) transport and mycolic acid biosynthesis, has emerged as a prominent target (3). As an essential component of cell wall biogenesis, MmpL3 has been validated for drug development through genetic studies, including transposon mutagenesis and CRISPRi knockdown, demonstrating its critical role in bacterial viability (4, 5). MmpL3 inhibitors span diverse chemical classes, including diamines, ureas, and indoles, and are frequently identified via pathway-independent phenotypic screens (2). These inhibitors mainly target transmembrane domains of MmpL3, disrupting proton-motive force (PMF)-driven TMM flipping and impairing cell wall integrity (6, 7). Resistance mutations cluster around key transmembrane residues, confirming MmpL3 as their primary target (8, 9). Experimental validation often employs both direct and indirect assays. While direct assays like SPR (10), proteoliposome-based proton transfer studies (11), and spheroplast assays (12) provide definitive evidence, indirect methods—such as lipid analysis (TMM accumulation and trehalose dimycolate [TDM] reduction) (13), reporter assays for cell wall stress (13, 14), or detection of lethal intracellular ATP burst (14, 15)—are commonly utilized due to their practicality. Together, these approaches have cemented MmpL3 as a high-value target for therapeutic intervention.

The 2,5,6-trisubstituted benzimidazoles (SBZ) are a class of antitubercular compounds, originally developed with a plausible hypothesis of targeting the cell division protein FtsZ (16). The SBZ compounds exhibit exceptional *in vitro* activity, with MICs in the nanomolar range, and effectively inhibit the growth of both drug-sensitive and drug-resistant Mtb strains (16–19). This compound series demonstrated great efficacy *in vivo*, achieving significant bacterial load reductions in murine models of acute Mtb infection, with outcomes comparable to or exceeding those of isoniazid, a frontline anti-TB drug (17–19). Optimizing the pharmacokinetics of the SBZ series has led to enhanced potency and pharmacological properties (18, 19). The series, containing over 500 synthesized compounds, was extensively explored in structure-activity relationship (SAR) studies (20, 21). However, validation of the target engagement is relatively weak, mostly based on a detection of FtsZ oligomers and an observation of the filaments formed *in vitro* (20, 22). With a battery of Mtb cell-based experiments, we have discovered that the mechanism of action (MoA) of SBZs should involve cell wall biosynthesis in mycobacteria. This activity underlines the effectiveness and highlights the promise of novel antitubercular agents.

## RESULTS

### Bacterial cytological profiling reveals that SBZ compounds show phenotypes similar to cell wall inhibitors

SBZ-A38 and 6B were selected from the literature as representatives of the whole series (18, 21). SBZ-A38 is one of their best lead compounds, with a thorough evaluation of its bactericidal killing and *in vivo* efficacy (18, 19), while SBZ-6B presents the best MIC as indicated by a recent publication (21). We synthesized the compounds using published protocols and verified them via nuclear magnetic resonance spectroscopy (NMR) and mass spectrometry (MS) (Fig. S1). We evaluated their MICs using a microplate-based Alamar Blue assay. SBZ-6B demonstrated an MIC of 0.3 µM against wild-type Mtb H37Rv, while the MIC of SBZ-A38 is 0.64 µM (Table 1). These values are consistent, within assay errors, with previously reported MICs of 0.078 µg/mL and 0.16 µg/mL, respectively. The SBZs are also effective against other slow-growing mycobacteria such as *Mycobacterium bovis* strain BCG.

**TABLE 1:** Structures and MICs of SBZ-6B and SBZ-A38

	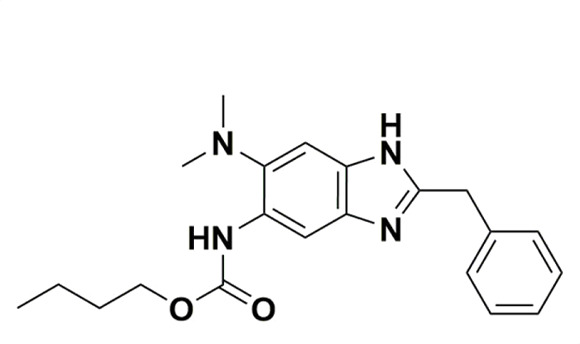	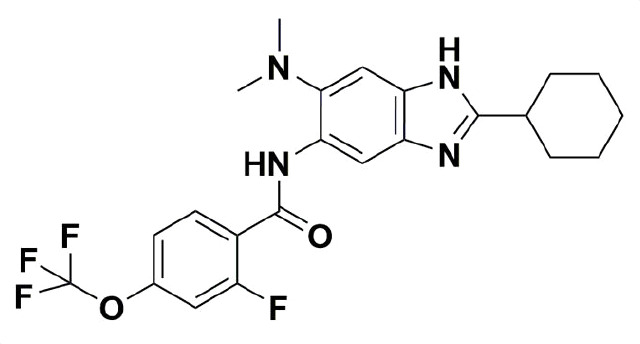
	SBZ-6B	SBZ-A38
MIC, *M. bovis*	0.21 ± 0.06 µM	0.32 ± 0.05 µM
MIC, *Mtb* H37Rv	0.3 ± 0.06 µM	0.64 ± 0.04 µM
MIC, *Mtb* H37Rv strain LP-0497754-RM301 (MmpL3 F255L, V646M, F644I)	0.76 ± 0.03 µM (2.5-fold)	1.5 ± 0.1 µM (2.3-fold)
MIC, *Mtb* H37Rv strain LP-mmpl3-7A (MmpL3 S591I)	4.8 ± 1.3 µM (16-fold)	3.8 ± 0.5 µM (5.9-fold)

Prior *in silico* analysis suggests that SBZ-A38 might bind close to the FtsZ T7 loop, which is critical for GTPase activity (19); however, subsequent modeling work with structural analogs showed a different docking pose (21). In our hands, neither SBZ-A38, SBZ-6B, nor the first-generation lead compound SBZ-P3G2 (Fig. S2A) at soluble concentrations up to 100 µM could inhibit or stimulate the GTPase activity of FtsZ when incubated with GTP (Fig. S2B) (23). In contrast, Zantrin Z2, which is a published small molecule inhibitor of Mtb FtsZ (24), and the non-hydrolyzable GTP analog GTPγS reproducibly inhibited this GTPase activity. Furthermore, we applied a biophysical thermal shift assay, aka differential scanning fluorimetry, to investigate the SBZ interaction with FtsZ (25); no change in protein melting temperature (Tm) was observed (Fig. S2C). In contrast, both Zantrin Z2 and GTPγS significantly affected the Tm of FtsZ, indicating that these two inhibitory compounds bind to the FtsZ protein, whereas the SBZs do not (Fig. S2C). We also attempted to generate resistant mutants using SBZ compounds, but all the efforts failed. Taken together, although SBZ compounds were reported to inhibit FtsZ polymerization *in vitro*, our results suggest that their potent bactericidal activity likely stems from targeting a different cellular pathway.

**Fig 1:**
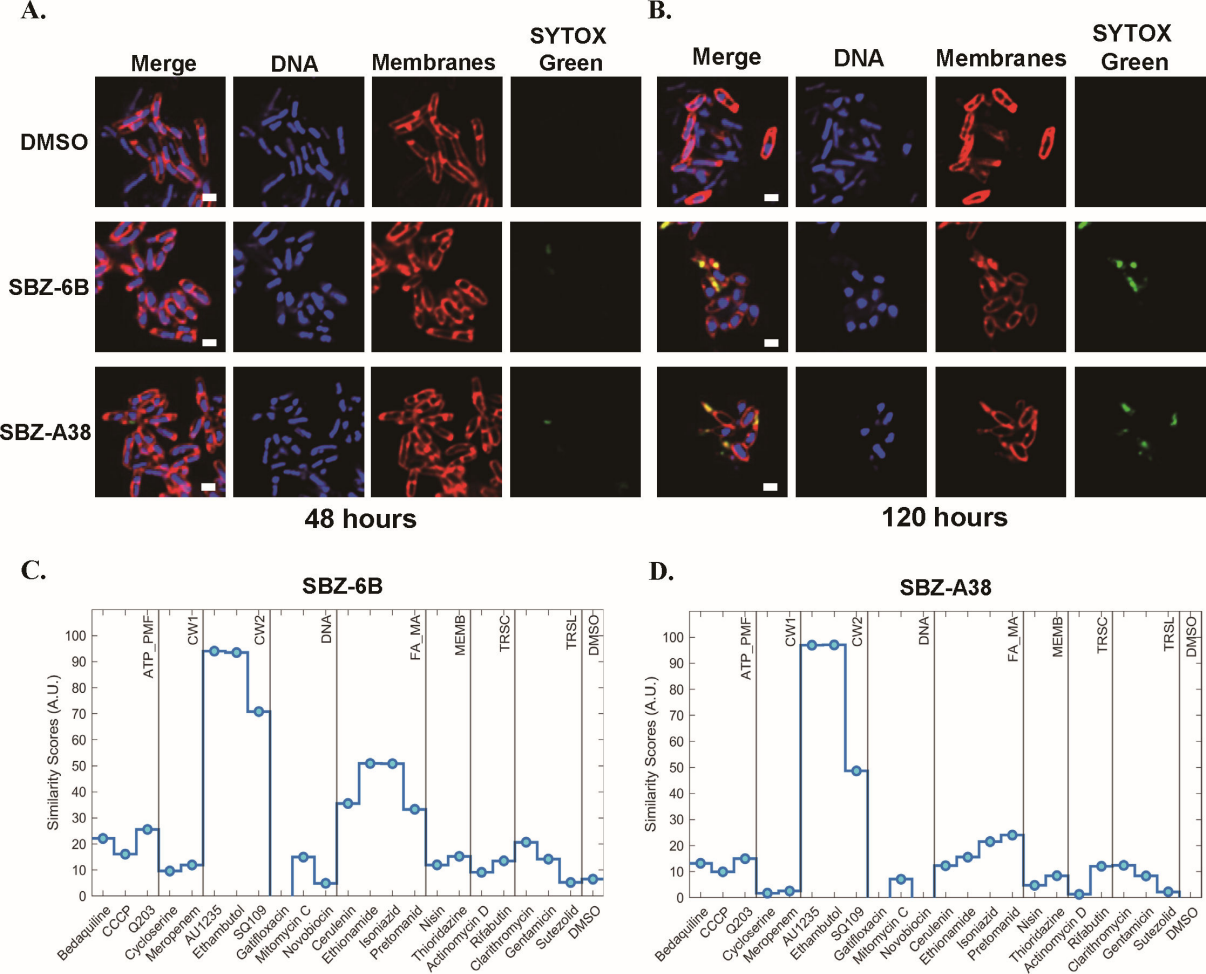
BCP of SBZ compounds. *M. tuberculosis* cells were challenged with 2% DMSO (top), SBZ-6B (middle), and A38 (bottom) at 2× MIC for 48 hours (**A**) and 120 hours (**B**), fixed with 16% paraformaldehyde, 8% glutaraldehyde, and 0.4 M phosphate buffer (pH 7.5). Cells were stained for 30 min with FM 4-64 (membrane; red), SYTO 40 (DNA; blue), and SYTOX Green (membrane impermeable DNA dye). Scale bars are 1 µm. (**C** and **D**) Plots of similarity scores for SBZ-6B (**C**) and A38 (**D**) to control compounds. There are nine possible predicted classifications including ATP synthase/PMF (ATP_PMF), cell wall (CW1 and CW2; affecting peptidoglycan and arabinogalactan/MmpL3 respectively), DNA, fatty acid/mycolic acid (FA_MA), membrane (MEMB), transcription (TRSC), translation (TRSL), and DMSO (negative control).

To investigate the cellular phenotype of SBZ-A38 and SBZ-6B inhibition, we employed the bacterial cytological profiling (BCP) technique (14, 26). *M. tuberculosis* mc^2^6206 cells were treated with compounds at 2× and 5× their MICs for 48 and 120 hours, followed by fixation and staining for membrane integrity, DNA, and cell structure (Fig. 1A and B). These treatments were performed at higher cell densities than those used during MIC determination to ensure sufficient cell numbers for microscopy; correspondingly, higher drug concentrations were required to observe cytological effects under these conditions. Bacteria exposed to SBZ compounds exhibited a rounded or ovoid shape compared to the rod-shaped morphology of the dimethylsulfoxide (DMSO)-treated control. Additionally, increased cell permeability was observed at both concentrations, as indicated by dye uptake. Despite these morphological changes, the cell length of SBZ-treated bacteria remained similar to that of the DMSO-treated control group, suggesting no statistically significant effect on cell division (Fig. S3). When compared to a set of compounds with known MoA, SBZ-treated cells showed the closest phenotypic similarity to a group of cell wall inhibitors that include AU1235, SQ109, and ethambutol (EMB; Fig. 1C and D). Notably, AU1235 and SQ109 are the well-characterized inhibitors of MmpL3. The observations strongly suggest that SBZ compounds might target this specific pathway of cell wall biosynthesis in Mtb.

### Whole-cell profiling assays demonstrate that SBZ compounds show similar phenotypes to MmpL3 inhibitors

**Fig 2:**
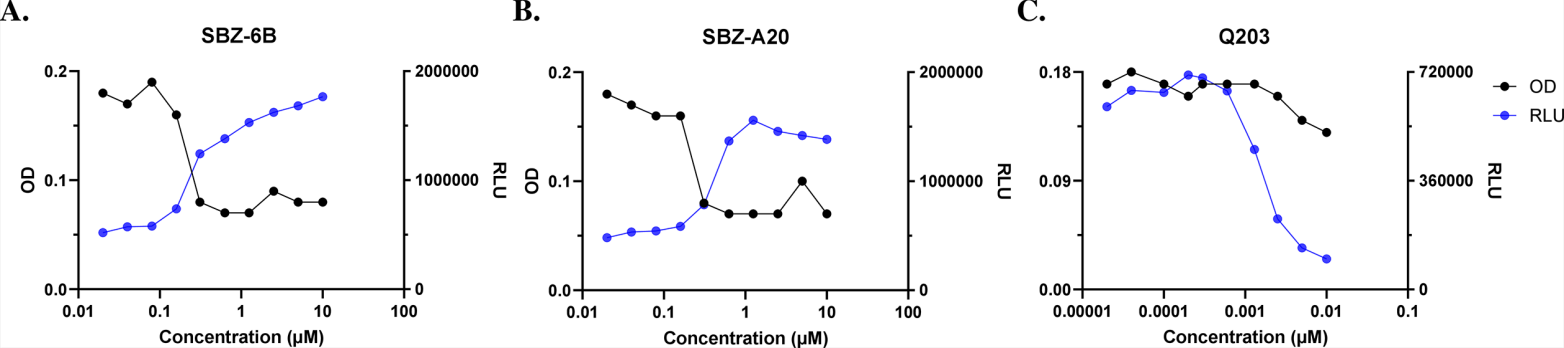
SBZ-6B and A38-induced ATP boost in Mtb. The cells were cultured to the logarithmic phase and challenged with compounds for 24 h. ATP was measured using the BacTiter-Glo reagent kit after incubation for 10 min and measuring relative luminescence units (RLU). Bacterial growth was determined after 5 days by reading OD_590_ and normalized to the DMSO control. (**A**) SBZ-6B treatment. (**B)** SBZ-A20 treatment. (**C**) Control compound Q203 treatment.

To further explore the MoA of SBZ compounds, we conducted a series of whole-cell profiling assays to examine how the bacterial physiology is impacted when the cells are treated with SBZs in comparison to other anti-Mtb compounds with known mechanisms, including cell wall synthesis inhibitors. Cell wall inhibitors can induce intracellular ATP accumulation due to decreased metabolic demands in dying cells (15). To investigate whether SBZs elicit a similar response, we quantified intracellular ATP levels in Mtb cells treated with SBZ-6B and SBZ-A38 for 24 hours (Fig. 2). Cell growth was monitored after 5 days. As expected, the control compound Q203, an inhibitor of the electron transport chain (27), caused a significant reduction in the ATP level (Fig. 2; Fig. S4). Results were normalized based on compounds’ MIC for better interpretation of comparison across compounds. In contrast, SBZ compounds induced a concentration-dependent ATP increase that is independent of cell density, supporting the hypothesis that they could disrupt cell wall biosynthesis (Fig. 2A and B). To benchmark these observations, we referenced control compounds previously established in the literature. Specifically, cell wall-targeting agents such as isoniazid (INH) and ethambutol (EMB) have been shown to cause a significant ATP increase through enhanced oxidative phosphorylation, whereas inhibitors of protein, RNA, or DNA synthesis (e.g., rifampicin [RIF]) do not exhibit this phenotype (15). These results provide a strong mechanistic foundation for interpreting the SBZ-induced ATP increase. 

**Fig 3:**
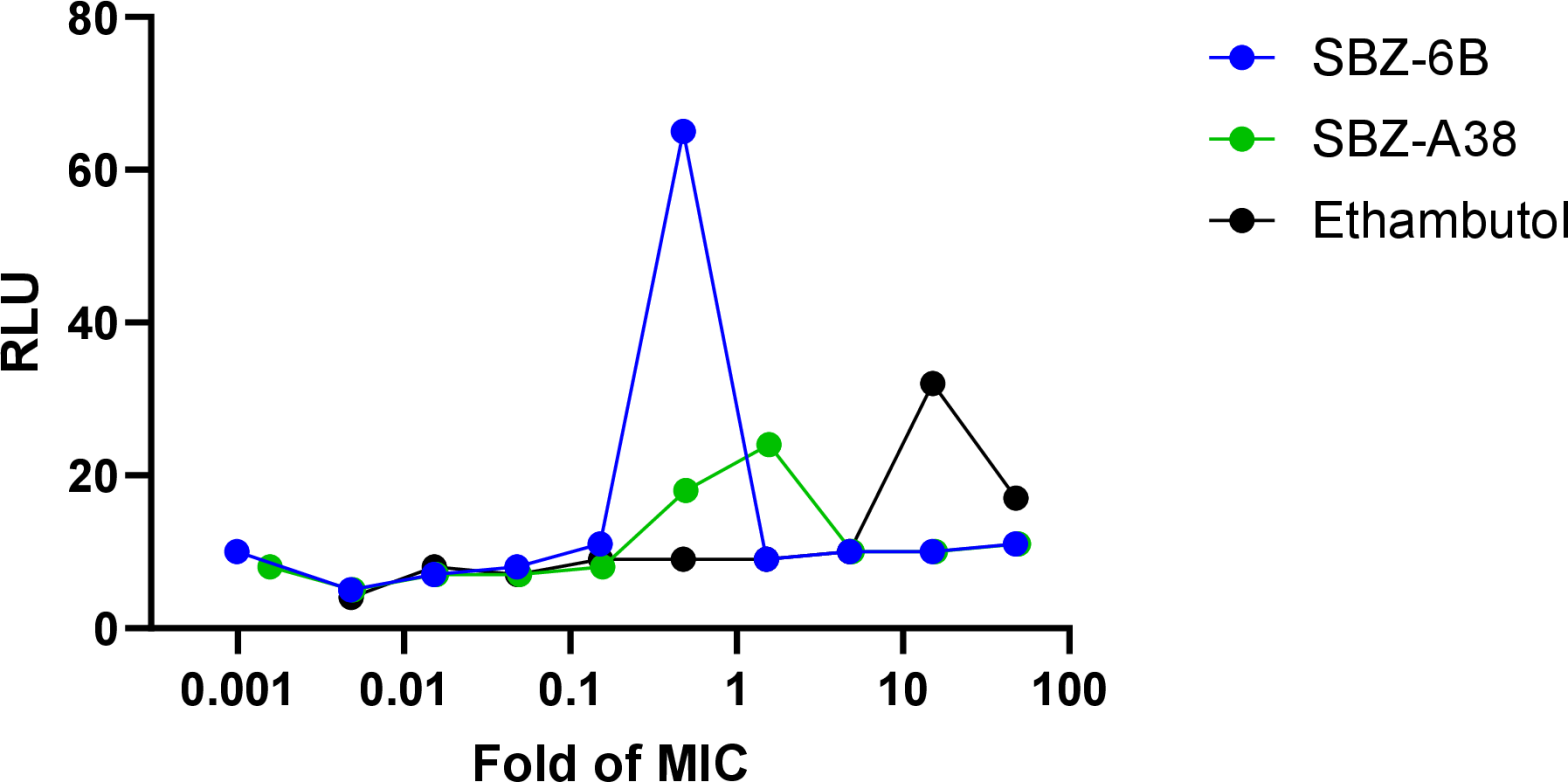
SBZ-6B and A38-induced cell wall stress. Mtb strain carrying PiniB–Lux reporter was cultured to mid-logarithmic phase and exposed to compounds for 3 days. Luciferase activity was measured after the addition of luciferin by measuring relative luminescence units (RLU).

Many cell wall inhibitors, including MmpL3 inhibitors, induce cell wall stress that can be quantified by measuring the expression level of the *iniBAC* operon (28, 29). Therefore, it is reasoned that exposure to SBZ compounds might stimulate cell wall stress. We utilized a luciferase reporter strain and exposed Mtb cells to serial dilutions of the compounds for 3 days (Fig. 3; Fig. S5). Similarly, results were normalized to multitudes of MICs to ensure consistent comparability. At concentrations near the MIC, significantly increased luciferase activity is observed for both SBZ-6B and SBZ-A38, indicating strong *iniBAC* promoter induction and cell wall stress. However, unlike the positive control EMB, which showed *iniBAC* activation only at concentrations well above its MIC, SBZ compounds triggered robust induction near their respective MICs, probably reflecting the different levels of impact of the inhibitions on cell wall biosynthesis. These results are consistent with previous reports showing that cell wall inhibitors stimulate *iniBAC* expression in parallel with an ATP burst (15).

**Fig 4:**
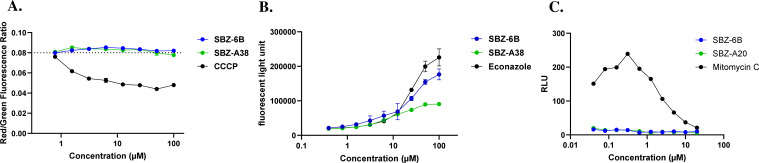
Whole-cell phenotypic assay of SBZ-6B and A38. (**A**) SBZ compounds did not induce changes in membrane potential. DiOC_2_(3) labeled *M. bovis* cells were exposed to compounds, and red/green (610 nm/535 nm) ratio was calculated to quantify membrane potential. (**B**) SBZ compounds induced reactive oxygen species (ROS) accumulation. 2′,7′-Dichlorofluorescin diacetate (DCFDA)-labeled *M. bovis* cells were incubated with compounds, and the fluorescent light at Ex485/Em535 was measured to record ROS production. (**C**) SBZ compounds did not induce DNA damage.

Membrane potential (ΔΨ) is another critical parameter of bacterial physiology. Change in ΔΨ is often associated with PMF disruption on transmembrane proteins, and genetically silencing *mmpL3* leads to a significant increase in ΔΨ (9). To determine if SBZ compounds affect ΔΨ, we used the ΔΨ-sensitive dye 3,3′-diethyloxacarbocyanine iodide (DiOC_2_[3]), which shifts fluorescence from red to green as ΔΨ changes. In our positive control, the PMF uncoupler carbonyl cyanide m-chlorophenylhydrazone, a dose-dependent shift in the red-to-green fluorescence ratio was observed, consistent with PMF collapse. However, neither SBZ-6B nor SBZ-A38 caused measurable changes in fluorescence, indicating that the compounds do not significantly affect ΔΨ (Fig. 4A). This observation aligns with prior findings that only a subset of MmpL3 inhibitors, such as SQ109, cause PMF disruption, while others do not (9).

Although not necessarily related to bactericidal effect, general stress responses, such as induction of reactive oxygen species (ROS), are observed in many bactericidal antibiotics (30, 31). It has been reported that mutations in transmembrane protein transporters are hypersensitive to oxidative stress (32). Furthermore, MmpL3 depletion in bacteria causes downregulation of genes involved in energy production and respiration (33). Therefore, we assessed whether SBZ compounds induce ROS production. Using a fluorescent reporter dye 2′,7′-dichlorofluorescin diacetate (DCFDA), we found that both SBZ compounds caused a concentration-dependent increase in ROS levels, similar to the positive control econazole (Fig. 4B). Econazole was selected as a control compound due to its well-established ability to induce ROS rapidly and reproducibly in Mtb (30). However, since this induction is observed at orders of magnitude above the MIC, we believe the accumulation we observed is not related to its bactericidal activity.

Finally, we examined whether SBZ compounds cause DNA damage since ROS-induced cell lethality is often associated with DNA strand breaks (34, 35). DNA damage activates the *recA* gene, which is involved in repairing single- and double-stranded breaks. Using a transcriptional reporter to measure recA promoter expression, we found that the positive control mitomycin C induced strong P_recA_ activation. In contrast, neither SBZ-6B nor SBZ-A38 showed detectable P_recA_ expression, indicating no detectable DNA damage (Fig. 4C).

In summary, SBZ-6B and SBZ-A38 exhibit similar physiological effects on Mtb cells that resemble those caused by MmpL3 inhibitors. Similar to many cell wall inhibitors (14, 15, 36), SBZ compounds elevate intracellular ATP levels and induce significant cell wall stress, but changes in membrane potential are not observed. While ROS generation was observed, the lack of DNA damage and a high MIC threshold for the induction suggest that it is a secondary response rather than a direct contributor to SBZ bactericidal activity. These phenotypes closely resemble those of established MmpL3 inhibitors. Although AU1235 was not included in all experimental assays in this study, it has been extensively profiled as a selective and potent MmpL3 inhibitor in previous work (37), and its well-defined behavior across multiple profiling platforms provides a reliable reference framework for interpreting the activity of SBZ compounds. These findings suggest that the potent bactericidal activity of SBZ compounds mainly arises from their ability to disrupt cell wall biosynthesis.

### SBZ’s MIC shifts in Mtb strains with regulated MmpL3 expression or MmpL3 mutations

**Fig 5:**
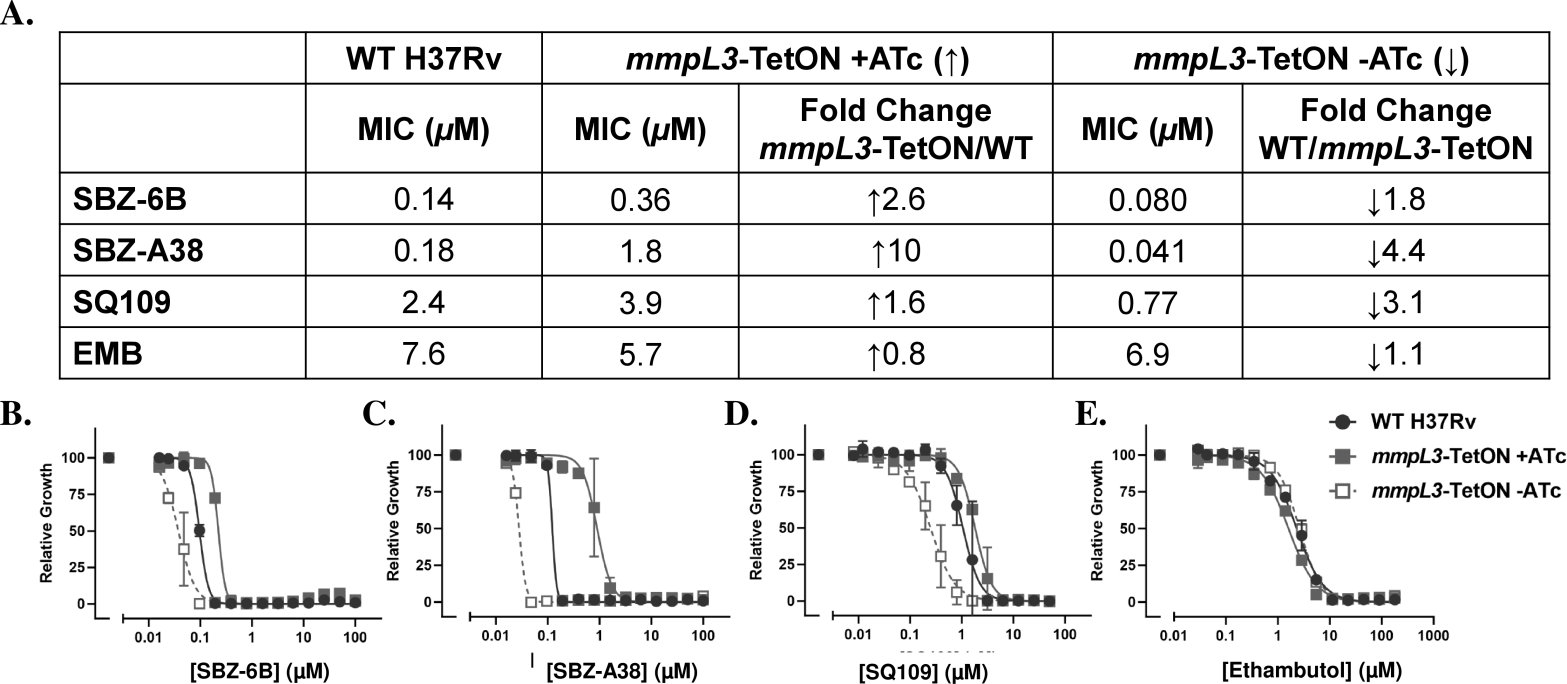
The MIC shifting of SBZ-6B and A38 in the *mmpL3*-TetON strain. (**A**) The calculated MICs and fold changes. (**B–E**) SBZ compound profiles against the engineered MmpL3 strain under over- (*mmpL3*-TetON + ATc) and under-expression (*mmpL3*-TetON-ATc) conditions. Strains were challenged with compounds for 14 days, and OD_580_ was measured as readout. SQ109 and EMB are chosen as positive and negative controls.

**Fig 6:**
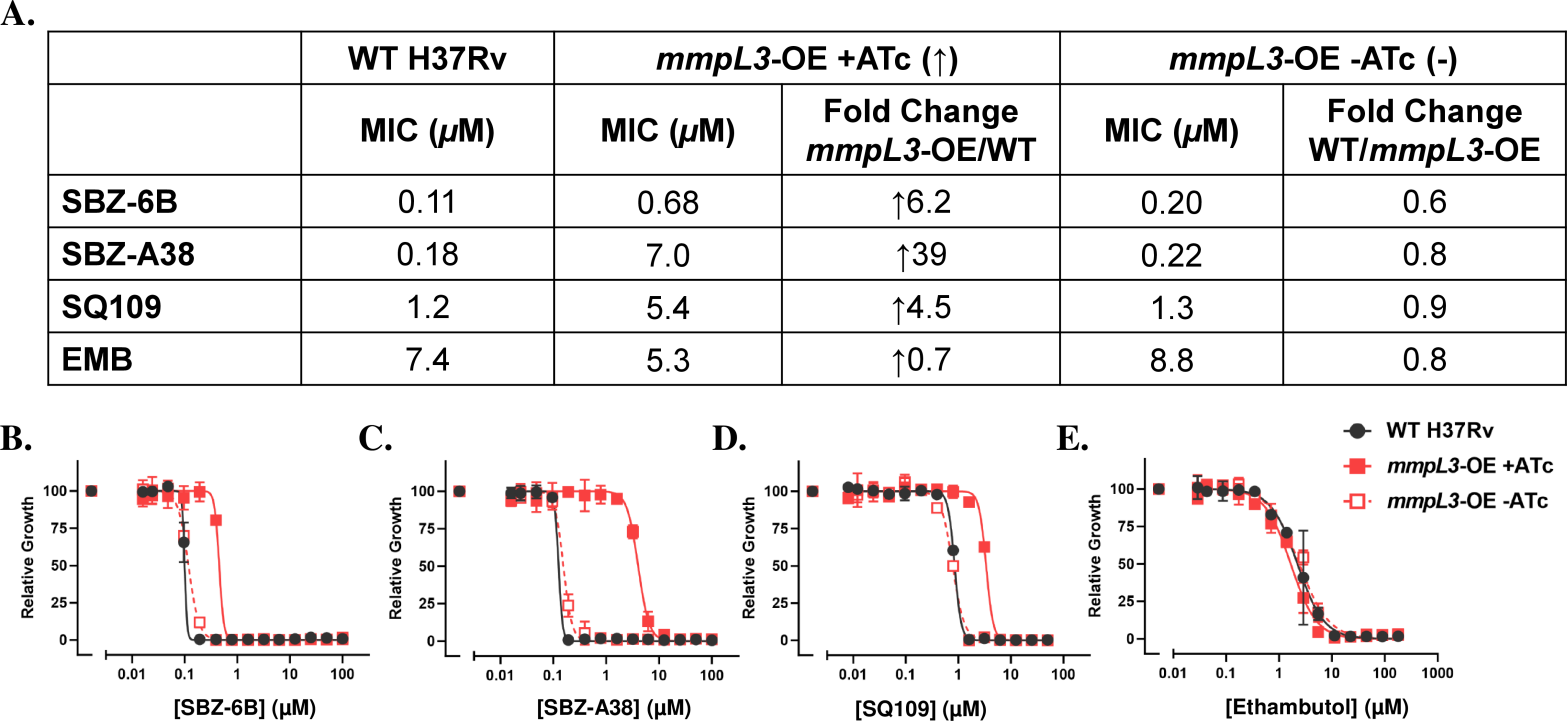
The MIC shifting of SBZ-6B and A38 in the mmpL3-OE strain. (**A**) The calculated MICs and fold changes. (**B–E**) SBZ compound profiles against this engineered MmpL3 strain under overexpression (*mmpL3*-OE + ATc) condition (↑) or not (-). Strains were challenged with compounds for 14 days, and OD_580_ was measured as a readout. SQ109 and EMB are chosen as positive and negative controls.

Our BCP data suggest that SBZ compounds share an MoA similar to known MmpL3 inhibitors such as AU1235 and SQ109. Additionally, whole-cell profiling assays confirmed that SBZ-treated Mtb cells exhibit phenotypes consistent with those observed for some established cell wall/MmpL3 inhibitors. To further investigate whether SBZ compounds target MmpL3, we utilized two transcriptionally regulated Mtb strains: *mmpL3*-TetON and *mmpL3*-OE (38). The first strain, *mmpL3*-TetON, allows bidirectional regulation of MmpL3 protein expression through anhydrotetracycline (ATc). In this system, MmpL3 is overexpressed when ATc is added, while depletion of MmpL3 occurs in the absence of ATc (38). The second strain, *mmpL3*-OE, overexpresses MmpL3 in the presence of ATc, but its expression reverts to physiological levels in the absence of the inducer. We tested the susceptibility of SBZ-6B and SBZ-A38 against these strains and compared their MICs to those of wild-type H37Rv. SQ109 and EMB served as positive and negative controls, respectively (Fig. 5 and 6). The assay was performed once using three technical replicates per condition, and results were consistent across replicates.

In the *mmpL3*-TetON strain, SBZ-6B exhibited a 2.6-fold increase of MIC in the presence of ATc and a 1.8-fold decrease in its absence, compared to wild-type (WT) H37Rv (Fig. 5A and B). Similarly, SBZ-A38 demonstrated a bidirectional MIC shift of 10-fold decrease and 4.4-fold increase under these conditions (Fig. 5A and C). In the *mmpL3*-OE strain, upregulation of MmpL3 in the presence of ATc resulted in 6.2- and 39-fold MIC increases for SBZ-6B and SBZ-A38, respectively (Fig. 6A through C). These trends were consistent with the results observed for the positive control SQ109, where its potency shifts corresponded to changes in the regulated MmpL3 expression (Fig. 5D and 6D). In contrast, the negative control compound EMB displayed no significant MIC variation during MmpL3 regulation (Fig. 5E and 6E).

To further evaluate the interaction between SBZ compounds and MmpL3, we determined their activity against two strains of Mtb with mutations in *mmpL3* associated with resistance to known MmpL3 inhibitors. Strain LP-0497754-RM301 has three mutations in MmpL3 (F255L, V646M, and F644I), which confer resistance to multiple MmpL3 inhibitor scaffolds, including AU1235 and spirocycles (9, 14). This strain demonstrated low-level resistance to SBZ compounds with a two- to threefold MIC shift (Table 1). Strain LP-Mmpl3-7A, containing a single S591I mutation obtained in another study (9), demonstrated high-level resistance to SBZ-6B and SBZ-A38 (16-fold and 5.6-fold increases, respectively [Table 1]). This further confirmed MmpL3 as the target. The resistance conferred by the mutations indicates that the four mutations in MmpL3, especially S591I, likely alter the binding of SBZ compounds, resulting in reduced potency. The observation links the SBZ’s MoA to a structural change in the MmpL3 protein.

Interestingly, the observed resistance mutations align partially with those reported for other benzimidazole-based MmpL3 inhibitors (3, 39). For instance, the activities of HC2099 and HC2184 are impacted by the S591I mutation, while the isolation of EJMCh4- and EJMCh6-resistant mutants did not find MmpL3 mutations tested in this study. The different activity profiles between SBZs and other benzimidazole inhibitors may result from variations in their chemical subgroups, particularly substitutions on the benzimidazole ring, which could affect their binding positions within the central proton transport channel of MmpL3 (39). This suggests that while SBZ compounds interact with MmpL3 similarly to some other benzimidazoles, their binding mode may be distinct from others, providing valuable insights into optimizing this chemical class. Overall, we have demonstrated the two SBZ compounds to be an MmpL3 inhibitor with bidirectional MIC shifts observed in MmpL3 over- and under-expressed strains, as further evidenced by cross-resistance toward previously reported MmpL3-resistant mutants.

### Molecular docking suggests that SBZ compounds’ binding mode of SBZ-6B may be similar to other MmpL3 inhibitors

**Fig 7:**
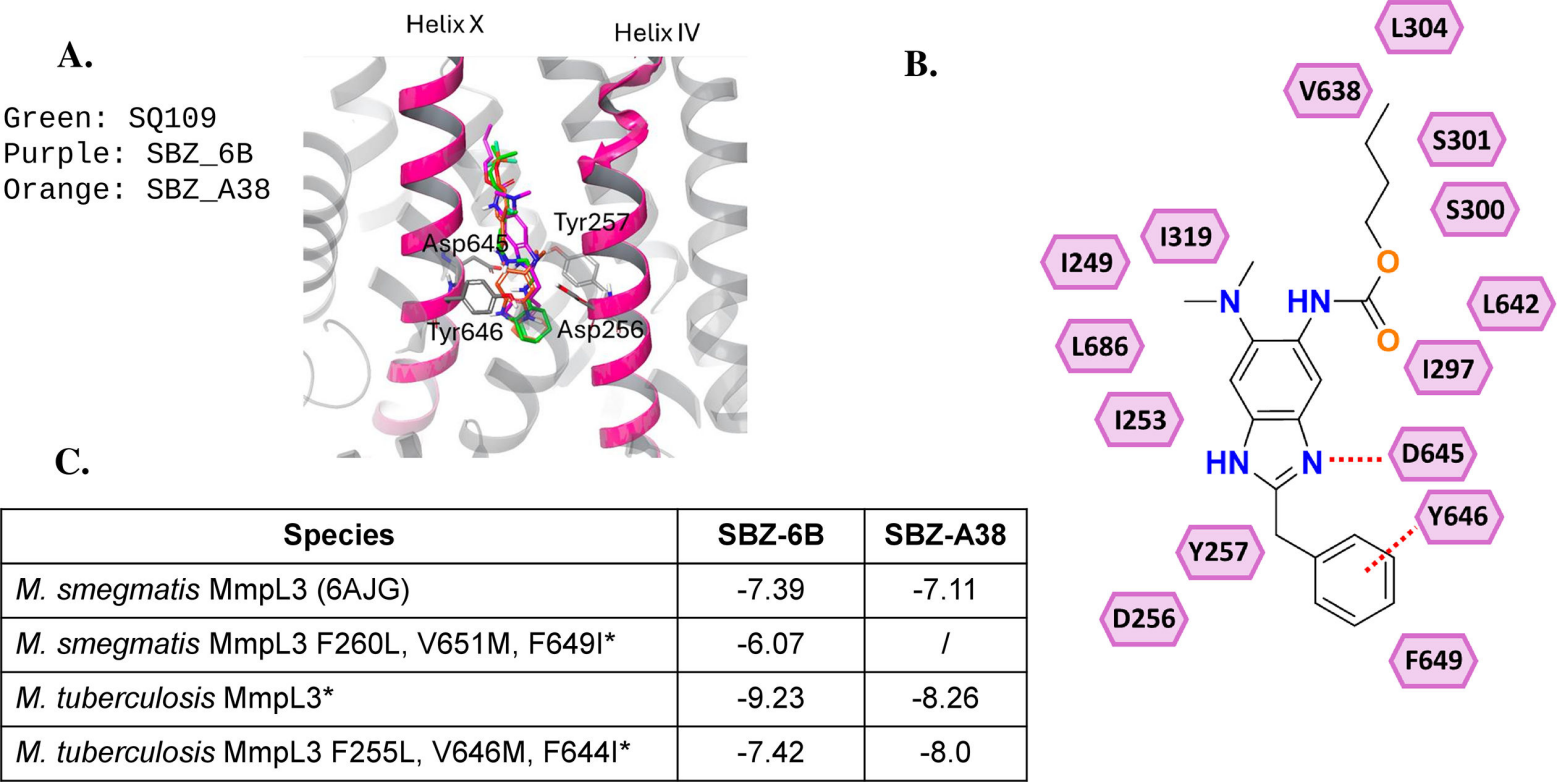
Molecular docking and interaction diagram of SBZ-6B and A38 in *M. smegmatis* MmpL3. (**A**) Docking models predicted are based on 6AJG. (**B**) Schematic representation of MmpL3-SBZ-6B interactions. (**C**) Docking score of SBZ-6B and A38 to *M. smegmatis* MmpL3 as well as a predicted (*) *M. tuberculosis* MmpL3.

To investigate the potential binding of SBZ compounds to MmpL3, we conducted a molecular docking study (Fig. 7), using the structure of *Mycobacterium smegmatis* MmpL3 in complex with SQ109 (PDB: 6AJG), given its high relevance to previously characterized MmpL3 inhibitors. Molecular docking was performed with Glide, and the protein and ligand were prepared and visualized with Maestro. Many known MmpL3 inhibitors possess a hydrogen bond donor that interacts with the conserved Asp645/Tyr646 residue pair on transmembrane helix 10, located within the core of the proton translocation channel (6). This interaction is critical for their binding and inhibitory activity. The best docking pose of SBZs revealed similar features (Fig. 7A). Specifically, the NH group on the benzimidazole ring forms a hydrogen bond with Asp645, while the adjacent aromatic ring engages in a π-π stacking interaction with Tyr646 (Fig. 7A and B). These interactions closely resemble the binding mode of SQ109 and other validated MmpL3 inhibitors, suggesting a shared mechanism of binding. The calculated docking scores for SBZ-6B and SBZ-A38 against 6AJG were −7.39 kcal/mol and −7.11 kcal/mol, respectively (Fig. 7C). Given our findings of MmpL3 mutations that confer resistance to SBZ compounds, we developed a triple mutation model (F260L, V651M, and F649I in *M. smegmatis*) based on 6AJG, which yielded a docking score of −6.07 kcal/mol for SBZ-6B (Fig. 7C). Unexpectedly, we could not dock SBZ-A38 to this triple mutant, likely due to the bulky benzo-trifluoride group of the benzimidazole ring. We hypothesize that this docking failure may also stem from structural differences between *M. smegmatis* MmpL3 and *M. tuberculosis* MmpL3. Consequently, we reformulated our model using a predicted structure of *M. tuberculosis* MmpL3 based on 6AJG. The updated model demonstrated a better binding affinity for SBZ-6B and A38, achieving docking scores of −9.23 and −8.26 kcal/mol, respectively. For the corresponding mutant (F255L, V646M, and F644I) of the Mtb MmpL3 model, the calculated docking scores against SBZ-6B and A38 were −7.42 and −8.0 kcal/mol, respectively. The relative differences in the docking scores could partially explain the experimental data that the MmpL3 triple mutant exhibits only a mild level of resistance to SBZ compounds.

This three-dimensional similarity of SBZ-6B and A38, as well as other known MmpL3 inhibitors (6, 40) in binding interactions, reinforces the hypothesis that SBZ compounds target MmpL3 by binding to its proton translocation function. Furthermore, the molecular interactions revealed that the SBZs could guide future optimization of the trisubstituted benzimidazole scaffold, enabling the development of more potent and selective MmpL3 inhibitors.

## DISCUSSION

MmpL3 has emerged as a promiscuous target for antitubercular drug discovery, with over 30 chemical scaffolds identified inhibiting its function. These scaffolds span diverse chemical classes and exhibit distinct modes of interaction with MmpL3, suggesting the protein’s dynamic flexibility and druggability (2). While it is broadly susceptible to the inhibition of many synthetic compounds, MmpL3 remains indispensable for Mtb viability, as it mediates the transport of TMM during cell wall biosynthesis. The chemically and genetically validated essential role makes it a compelling therapeutic target.

In this study, we re-evaluated a series of trisubstituted benzimidazoles (SBZ compounds). This >500-compound series has demonstrated excellent MIC values, bactericidal activity, and significant bacterial reductions in a murine model of acute Mtb infection. By utilizing the most recent technology, BCP, we observed that SBZ-treated Mtb cells exhibit a phenotype similar to known MmpL3 inhibitors, such as AU1235 and SQ109, including cell wall stress and increased permeability. Additional whole-cell phenotypic assays further supported this notion, including *iniBAC* operon activation and intracellular ATP accumulation, ineffectiveness on membrane potential. Susceptibility testing in transcriptionally regulated *mmpL3* strains revealed significant MIC shifts in response to mmpL3 overexpression or knockdown, while strains with specific MmpL3 mutations (e.g., S591I) showed much-reduced sensitivity to SBZ compounds. Molecular docking studies confirmed interactions between SBZ compounds and the conserved Asp645/Tyr646 residues in the proton translocation channel of MmpL3 protein, resembling other MmpL3 inhibitors. Collectively, these findings strongly suggest that SBZ compounds target MmpL3 as the primary MoA, offering new insights into their activity and potential optimization.

Benzimidazole has an important role in medicinal chemistry as it is found in a wide range of pharmacologically active compounds. Benzimidazoles were previously found and explored to target MmpL3, including HC2099/MSU-43085 and EJMCh series (3, 39, 41). Like the SBZ, these series share features, such as potent MIC values, bactericidal activity against Mtb clinical strains, and significant efficacy in acute murine models comparable to isoniazid. Despite these shared characteristics, the structural optimization of these series has primarily focused on modifications at position 2 of the benzimidazole ring, with limited exploration of substitutions at positions 5 and 6. In contrast, the SBZ compounds in this study are the first reported to incorporate significant substitutions at position 6. The modifications have provided new insights into the SAR of benzimidazole-based MmpL3 inhibitors (16, 21). The new direction for the optimization of benzimidazole core modifications at these positions may enhance metabolic stability, improve pharmacokinetics, and potentially increase potency against resistant Mtb strains.

Our findings indicate that SBZ-6B and SBZ-A38 target MmpL3, as evidenced by BCP and susceptibility testing with mycobacterial strains bearing the regulated expression of *mmpL3* or known MmpL3 resistance-conferring mutations. However, additional biochemical experiments could be performed to strengthen the proposition of the action mechanism of SBZs. Functional assays such as lipid profiling (42) or TMM flippase/proton translocation studies (11, 12) would reveal the consequences of the inhibited MmpL3 activity at the cell level. It is also important to note that this study does not exclude the possibility that SBZ compounds have additional intracellular targets. Indeed, previous research has shown that some MmpL3 inhibitors interact with other cellular pathways. For example, SQ109 disrupts menaquinone biosynthesis by targeting MenA (43), and THPP has been shown to inhibit enoyl-CoA hydratase EchA6 in addition to MmpL3 (44). Similarly, although SBZ compounds were originally reported to affect FtsZ polymerization *in vitro*, our current biochemical and cell-based data do not support FtsZ as a primary or secondary target, nor do we have direct evidence that SBZs gain access to the cytoplasmic compartment. Indeed, the inability to isolate resistant mutants despite multiple attempts is notable, especially in contrast to the emergence of resistance observed with other MmpL3 inhibitors. While we were unable to isolate resistant mutants under our conditions, the confirmed resistance of the S591I mutant demonstrates that a single-step mutation in MmpL3 can confer reduced susceptibility. We speculate that this failure may be due to a combination of factors, including a steep fitness cost associated with resistance-conferring mutations in MmpL3 under SBZ pressure, or a distinct binding mode of SBZs that restricts mutational escape. These speculations are intriguing but require further validation. Additionally, the physicochemical characteristics of SBZ compounds may confer membrane-disruptive or polypharmacological activity that reduces the likelihood of single-locus resistance. While such activity may contribute to efficacy and reduce the frequency of resistance, it also raises concerns about off-target interactions and unmanageable cytotoxicity. These considerations might be addressed through comprehensive selectivity profiling. For example, the large trifluoromethoxy benzyl group on SBZ-A38 may bring about alternative antibacterial activity compared to SBZ-6B. These possibilities underscore the potential advantage of SBZs in resistance suppression and may inform future structure-based design strategies. Given these considerations, SBZ-6B represents a particularly suitable scaffold for further optimization centered on MmpL3, while also offering opportunities to explore secondary targets to enhance antibacterial efficacy and resilience to resistance. Subsequent steps could involve exploring the compounds from the large SBZ series (21) and employing our predicted MmpL3 docking structure to perform binding energy correlations. This approach will ultimately lead to a refined quantitative SAR docking model.

In conclusion, our study demonstrates that SBZ-6B and SBZ-A38, originally reported as FtsZ polymerization inhibitors, induce cellular phenotypes consistent with MmpL3 inhibition. Their on-target activity is supported by results from MmpL3-dosage and mutant strain assays. While MmpL3 appears to be a key target of SBZs, further experiments are needed to confirm whether additional pathways contribute to the compounds’ overall MoA. These findings establish SBZ compounds as promising candidates for antitubercular development targeting MmpL3 and provide a foundation for further optimization and mechanistic exploration.

## MATERIALS AND METHODS

### Bacterial strains, growth conditions, and reagents

*M. tuberculosis* and *M. bovis* BCG Str. Pasteur 1173P2 were cultured in 7H9 Middlebrook medium (Difco; supplemented with 10% oleic acid-albumin-dextrose-catalase, 0.5% glycerol, and 0.05% tyloxapol). SBZ-6B and A38 were synthesized according to the literature (17, 21). SBZ-P3G2 was synthesized and distributed by the Medicines for Malaria Venture as MMV1578842. Compounds were dissolved in DMSO to make a 20 mM stock solution. Cellular dyes FM 4-64, SYTO 40, and SYTOX Green were purchased from Invitrogen. DiOC_2_(3), DCFDA, and antibiotics were purchased from MedChemExpress (MCE). BacTiter-Glo microbial cell viability assay kit was purchased from Promega.

### Bacterial cytological profiling

Bacterial cytological profiling was conducted at Linnaeus Bioscience Inc (26). *M. tuberculosis mc^2^6206* H37Rv *ΔpanCD ΔleuCD* strain was cultivated at 30°C until reaching the stationary phase, serving as the parent culture. This parent culture was then diluted to OD_600_ of approximately 0.06–0.08 and incubated at 30°C for 18–20 hours. Following this, the bacteria were exposed to test compounds at 2× the MIC for a duration of 48–120 hours. After the exposure period, the cells were harvested and fixed using a solution consisting of 100 µL of 16% paraformaldehyde, 3 µL of 8% glutaraldehyde, and 20 µL of 0.4 M phosphate buffer (pH 7.5). The fixed cells were then washed twice with 7H9 broth and stained for visualization. The staining protocol included the use of SYTO 40 for DNA visualization, FM 4-64 for membrane staining, and SYTOX Green, a membrane-impermeant nucleic acid stain, for 30 min prior to processing for visualization. Phenotypic similarity was quantified with similarity scores (Fig. 1C and D) calculated as the average minimum distance to control compounds with known MoAs embedded in a multidimensional space and rescaled to a range from 0 to 100 (26).

### Intracellular ATP measurement

ATP was measured as described previously (14). In brief, H37Rv-LP (ATCC 27294) was grown to mid-log phase, diluted, and exposed to test compounds in 96-well plates at 37°C with 5% CO_2_ for 24 h. BacTiter-Glo reagent was added, incubated for 10 min at room temperature (RT), and luminescence measured. A parallel plate for measuring growth by OD_590_ was incubated at 37°C with 5% CO_2_ for 5 days.

### Cell wall stress assay with iniBAC reporter

The P_iniBAC_ reporter assay was performed as described previously (14, 29). H37Rv carrying P_iniBAC_-*lux* reporter plasmid was cultured in medium with 15 µg/mL kanamycin at 37°C for 7 days until reaching mid-log phase. The culture was adjusted to OD_590_ of 0.02 and added to 96-well plates containing test compounds. Plates were incubated at 37°C for 3 days, after which an equal volume of substrate solution (1 M HEPES buffer, pH 7.8; 1M dithiothreitol (DTT); and 10 mg/mL luciferin) was added. Plates were covered with aluminum foil, incubated at RT for 25 min and luminescence measured.

### Membrane potential measurement

Membrane potential measurement was performed as described (38). WT *M. bovis* strain was grown to OD_600_ = 0.6. Bacterial cells were harvested and resuspended in fresh 7H9 full medium containing 15 uM DiOC_2_(3) for 20 min and washed twice. The labeled bacteria culture is then calibrated to an OD_600_ of 0.8 and aliquoted to 96-well microtiter plates containing diluted test compounds. Plates were incubated at RT for 15 min. Fluorescence was measured at 488/530 nm (green) and 488/650 nm (red). The red/green (610 nm/535 nm) fluorescence intensity ratio was calculated and used to quantify membrane potential. Duplicates were performed for each dilution.

### Intracellular ROS measurement

Intracellular ROS was measured as described (15). WT *M. bovis* strain was grown to late-log phase (OD_600_ = 0.8–1.0). Bacterial cells were spun down and resuspended in fresh 7H9 full medium containing 40 uM dichlorodihydrofluorescein diacetate and incubated at 37°C for 30 min. The fluorescent probe was then washed off, and bacteria were aliquoted to 96-well microtiter plates containing diluted test compounds and incubated for 2.5 hours. Green fluorescence (488/530) was measured to quantify the ROS level in bacterial cells. Duplicates were performed for each dilution.

### DNA damage assay

The P_recA_-*lux* reporter strain was grown to mid-log phase and then diluted to a starting OD_600_ of ∼0.05. Bacterial cells were then diluted and inoculated into 96-well microtiter plates and incubated together with serially diluted test compounds at 37°C with 5% CO_2_ for 3 days. A substrate solution containing luciferin is added, and the plates were incubated in the dark for 25 min. Luciferase activity (recorded as relative luminescence units) was then measured as readout.

### SBZ activity profiles with transcriptionally regulated mmpL3 strains

Two transcriptionally regulated strains of *mmpL3* (*mmpL3*-TetON and *mmpL3*-OE) and the parental H37Rv were initially grown to stationary phase, then diluted to OD_580_ of ~0.05 and grown at least 7 days until mid-log phase (in the presence of 500 ng/mL ATc and 25 µg/mL kanamycin for MmpL3-regulation strains) at 37°C with 5% CO_2_. Bacterial cells were then harvested and diluted to an OD_580_ of ~0.01 in 7H9 full medium supplemented with and without ATc and incubated with test compounds in triplicate in 384-well microtiter plates. Plates were incubated for 14 days, and OD_580_ was measured as readout. Triplicates were performed for each dilution.

### Molecular docking, quantification, and statistical analysis

Molecular docking was performed with Glide using the structure of *M. smegmatis* MmpL3 in complex with SQ109 (PDB: 6AJG). The protein and ligand were prepared and visualized with Maestro. The molecular docking process was systematically conducted in a tripartite fashion: preparation of the protein structure, readying of the ligands, and execution of the docking algorithm. The protein preparation phase was undertaken using the Schrödinger Protein Preparation Workflow panel (45), accessed through the Maestro interface (version 12.9.123, released in 2021.3), where default parameters were employed. This phase comprehensively included the assignment of appropriate bond orders, the introduction of hydrogen atoms, the preservation of water molecules exceeding a 5 Å threshold relative to ligands, and meticulous reconstruction of absent segments such as side chains and loop structures. Following this, hydrogen bond configurations were optimized in accordance with pKa predictions rendered by PROPKA. This was succeeded by a restrained minimization of the protein’s energy, utilizing the force field specifications provided by default. The generation of protein grid files was facilitated using the Receptor Grid Generation panel. The preparation of the ligands was executed via the command line instruction ligprep -ismi *.smi -omae *maegz, resulting in the conversion of SBZ-6B and SBZ-A38 from bidimensional SMILES formats into tridimensional structural models. Then, the elaborated structures were algorithmically docked into their anticipated binding sites by utilizing the Glide SP docking protocol. This docking employed the well-established OPLS_2005 force field and adhered to the default parameter set (46).

The docking model with the best binding affinity score was selected for further analysis. Graphic data were prepared and analyzed with GraphPad Prism software, and the error bars represent SEM. Statistical analysis was conducted using an unpaired two-tailed Student’s *t*-test, and significance is displayed as *P < 0.1 or **P < 0.01.

## SUPPLEMENTAL MATERIAL

Supplemental material aac.00368-25-s0001.pdf Fig. S1 to S5.

ASM does not own the copyrights to Supplemental Material that may be linked to, or accessed through, an article. The authors have granted ASM a non-exclusive, world-wide license to publish the Supplemental Material files. Please contact the corresponding author directly for reuse.
