# Supplementary material for: Microbiological evidence for the trisubstituted benzimidazoles targeting MmpL3 in Mycobacterium tuberculosis
Source: Antimicrob Agents Chemother. 2025 Aug 19;69(10):e00368-25. doi: 10.1128/aac.00368-25 (PMC12486814; doi:10.1128/aac.00368-25)
Supplement: Supplemental material — Fig. S1 to S5. [file aac.00368-25-s0001.pdf]

**A.**

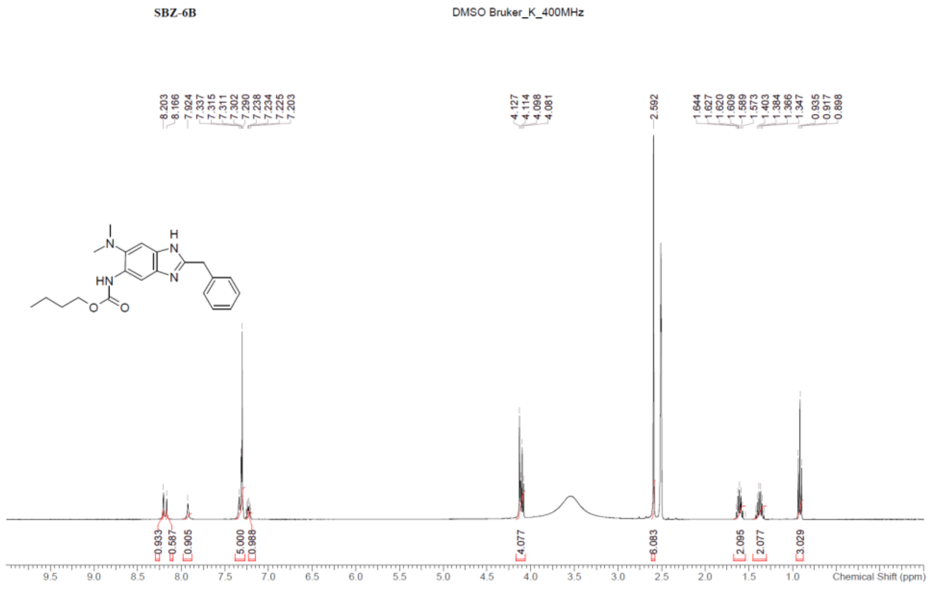


**SBZ-6B (butyl (2-benzyl-6-(dimethylamino)-1H-benzo[d]imidazol-5-yl)carbamate):** 1H NMR (400 MHz, DMSO) δ 8.20 (s, 1H), 8.17 (s, 1H), 7.92 (s, 1H), 7.35 – 7.25 (m, 5H), 7.24 – 7.19 (m, 1H), 4.15 – 4.05 (m, 4H), 2.59 (s, 6H), 1.66 – 1.55 (m, 2H), 1.45 – 1.30 (m, 2H), 0.92 (t, J = 7.2 Hz, 3H). MS (ESI) m/z 367.4 $[M+H]^+$.

**B.**

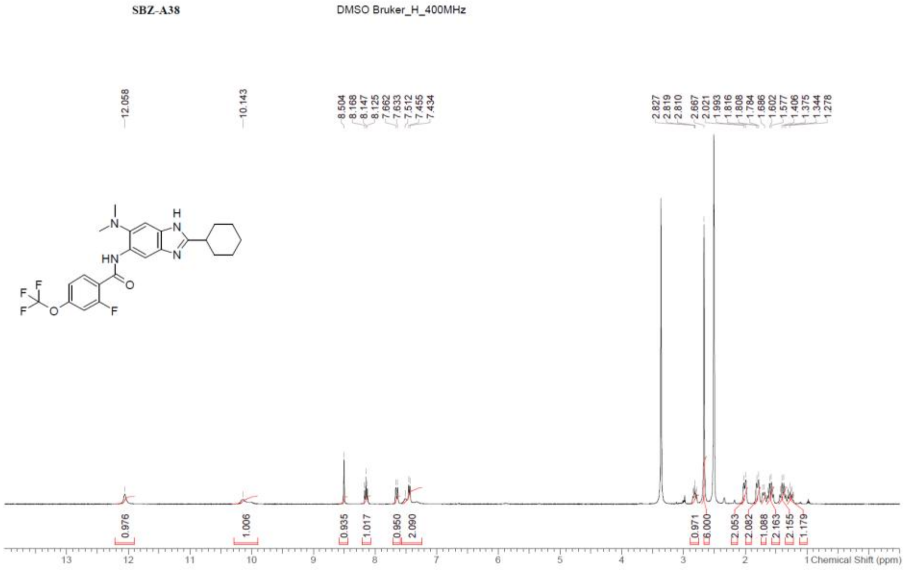


**SBZ-A38 (N-(2-cyclohexyl-6-(dimethylamino)-1H-benzo[d]imidazol-5-yl)-2-fluoro-4-(trifluoromethoxy) benzamide):** 1H NMR (400 MHz, DMSO) δ 12.06 (s, 1H), 10.14 (s, 1H), 8.50 (s, 1H), 8.15 (t, J = 8.4 Hz, 1H), 7.65 (d, J = 11.6 Hz, 1H), 7.46 – 7.41 (m, 2H), 2.83 – 2.75 (m, 1H), 2.67 (s, 6H), 2.05 – 1.95 (m, 2H), 1.85 – 1.79 (m, 2H), 1.78 – 1.69 (m, 1H), 1.65 – 1.55 (m, 2H), 1.45 – 1.30 (m, 2H), 1.29 – 1.20 (m, 1H). MS (ESI) m/z 465.1 [M+H]+.

**Figure S1.** 1H NMR and ESI-MS data of (A) SBZ-6B and (B) SBZ-A38.

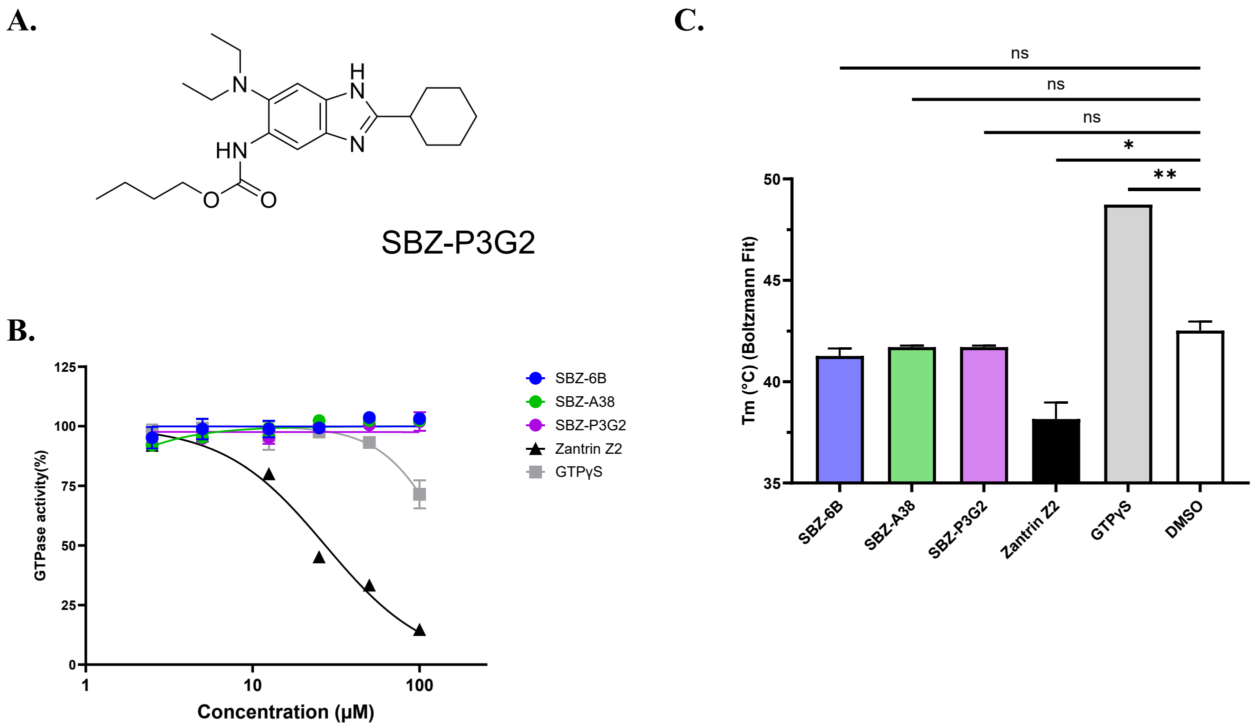


**Figure S2.** SBZ compounds did not affect the GTPase or change the melting temperature of FtsZ protein. A) Structure of SBZ-P3G2. B) Compound-preincubated FtsZ protein was incubated with GTP for 3 hours, then GTPase activity was measured via BIOMOL green reagent. C) Tm values of FtsZ in the presence of SBZ and control compounds. DMSO solvent control or 50 μM of compounds were incubated with FtsZ and SYPRO Orange dye and subject to a standard thermal shift assay. Tm was calculated based on Boltzmann derivative fit. Data points in duplicates are presented as an average with the error bar representing SEM. Statistical analysis is conducted using an unpaired two-tailed Student's t-test and significance is displayed as *P<0.1 or **P < 0.01.

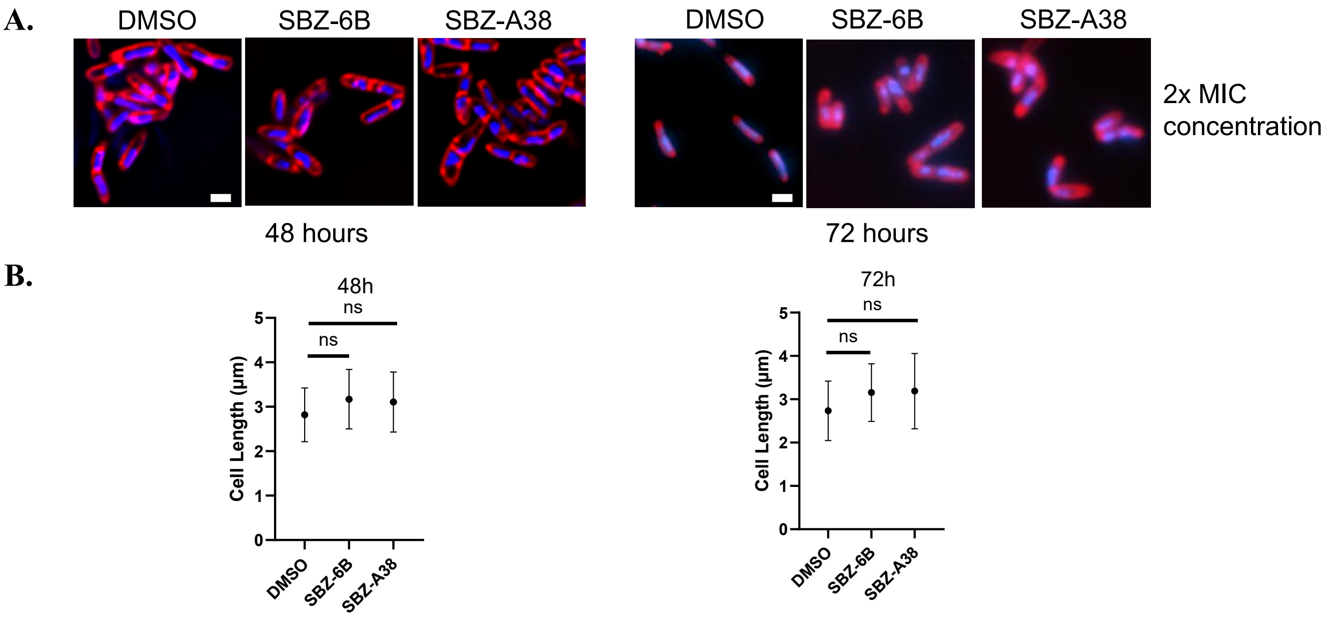


**Figure S3.** SBZ compounds-treated cells do not show increased cell length. A) *M.tuberculosis* cells were challenged with SBZ compounds or DMSO for 48 (left) or 72 hours (right), then fixed and stained with DAPI(blue) and nile red (red). B) Cells length was calculated with ImageJ. Data presented are an average of ~100 cells and error bars represent SD of the mean. Statistical analysis was conducted using an unpaired two-tailed Student's t test.

**A.**

**B.**

**C.**

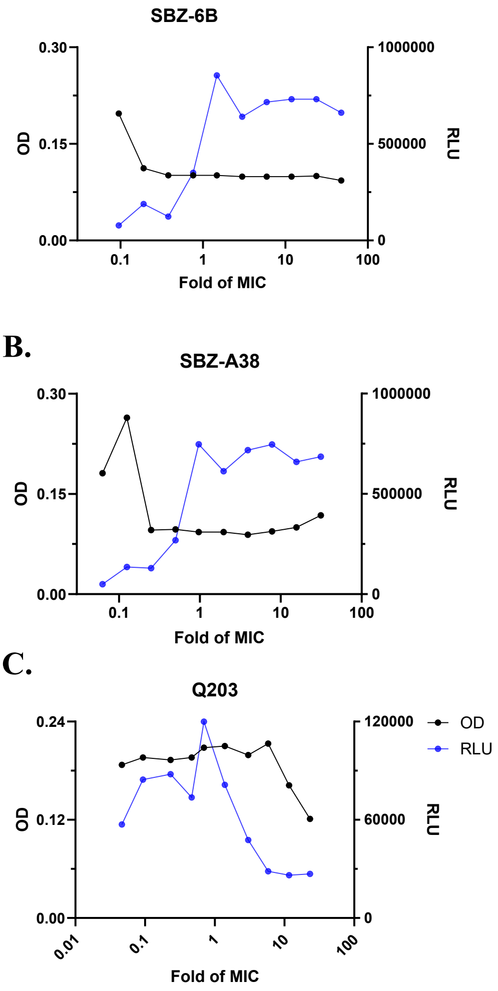


**Figure S4.** SBZ-6B and A38-induced ATP boost in Mtb (Assay replicate of Figure 2). The cells were cultured to the logarithmic phase and challenged with compounds for 24 h. ATP was measured using the BacTiter-Glo reagent kit after incubation for 10 min and measuring relative luminescence units (RLU). Bacterial growth was determined after 5 days by reading $OD_{590}$ and normalized to the dimethyl sulfoxide (DMSO) control.

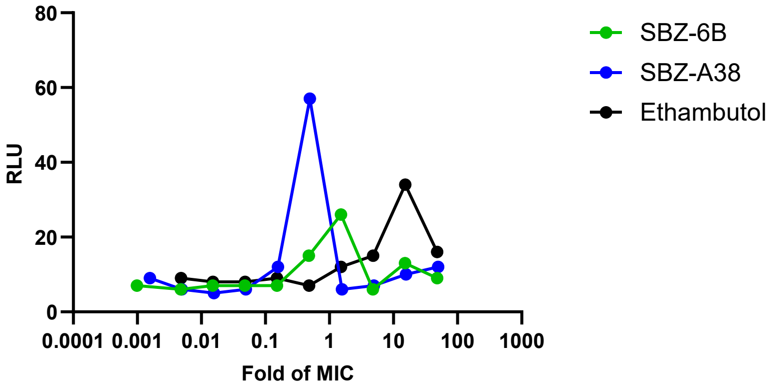


**Figure S5.** SBZ-6B and A38-induced cell wall stress (assay replicate of Figure 3). Mtb strain carrying $P_{iniB}$–Lux reporter was cultured to mid-logarithmic phase and exposed to compounds for 3 days. Luciferase activity was measured after the addition of luciferin by measuring relative luminescence units (RLU).
